# Intraocular Pressure-Lowering Efficacy of a Sustained-Release Bimatoprost Implant in Dog Eyes Pretreated with Selective Laser Trabeculoplasty

**DOI:** 10.1089/jop.2021.0104

**Published:** 2022-05-03

**Authors:** Corine Ghosn, Lakshmi Rajagopalan, Sveti Ugarte, Shruti Mistry, Werhner Orilla, Margot L. Goodkin, Michael R. Robinson, Michael Engles, Mohammed Dibas

**Affiliations:** Allergan, An AbbVie Company, Irvine, California, USA.

**Keywords:** bimatoprost, glaucoma, intraocular pressure, laser trabeculoplasty, ocular hypertension, sustained-release implant

## Abstract

**Purpose:**

To assess the intraocular pressure (IOP)-lowering effect of a biodegradable bimatoprost implant following selective laser trabeculoplasty (SLT) in a canine model.

**Methods:**

Unilateral SLT was performed in 11 normotensive, treatment-naive beagle dogs. IOP was measured at baseline (pre-SLT) and weekly post-SLT (≤10 weeks). After IOP returned to baseline or at 10 weeks (whichever occurred first), a sustained-release bimatoprost implant was administered bilaterally in the anterior chamber of each animal. IOP was measured weekly for 4 weeks and then every 2 weeks up to week 42.

**Results:**

The main outcomes included the IOP change (%) from baseline, calculated in both eyes in the overall population, SLT responder subgroup (defined by peak IOP reduction from baseline ≥3 mmHg or ≥15% for >1 week post-SLT), and SLT nonresponder subgroup (defined by peak IOP reduction from baseline <3 mmHg or <15%). The bimatoprost implant lowered IOP similarly in both the SLT-treated and fellow SLT-naive eyes. Following bimatoprost implant administration, the mean (standard deviation [SD]) peak IOP reduction from baseline was 34.4% (8.5%) in SLT-treated eyes and 35.7% (5.9%) in fellow SLT-naive eyes. The bimatoprost implant lowered IOP comparably (*P* > 0.17) in eyes that responded to SLT (mean [SD] peak IOP reduction, 34.6% [10.7%]; *n* = 6) and those that did not (mean [SD] peak IOP reduction, 34.1% [6.1%]; *n* = 5).

**Conclusion:**

The bimatoprost implant effectively lowered IOP in eyes pretreated with SLT, regardless of response to SLT. The current data suggest that eyes previously treated with SLT can still benefit from the intracameral bimatoprost implant.

## Introduction

Glaucoma refers to a group of irreversible, chronic/progressive diseases characterized by retinal ganglion cell degeneration and changes in the optic nerve head^1^ that can lead to vision loss and blindness if left untreated.^2,3^ Open-angle glaucoma (OAG) is the most common form worldwide, and risk factors include older age and high intraocular pressure (IOP), among others.^3^

For patients with glaucoma or ocular hypertension (OHT, i.e., high IOP without nerve damage), lowering IOP remains the only means to prevent disease progression, and due to their efficacy, once-daily dosing, and safety/tolerability profile, ophthalmic solutions containing prostaglandin analogs/prostamide (PGAs), such as bimatoprost and latanoprost, are often used as first-line IOP-lowering therapy.^2,3^ To further increase convenience of treatment with an established IOP-lowering agent, the bimatoprost implant 10 μg (DURYSTA™; Allergan, an AbbVie company) was developed as an intracameral, biodegradable implant designed to release bimatoprost for 3–4 months.^4–6^

In a prospective, dose-ranging, paired-eye, 24-month, controlled, phase 1/2 study, the bimatoprost implant showed favorable safety and efficacy profiles in OAG across the range of dose strengths tested.^4^ In phase 3 evaluation, bimatoprost implants of the 10- and 15-μg dose strengths effectively decreased IOP in patients with OAG or OHT,^7^ and the 10-μg dose strength implant (DURYSTA™) was recently approved for a single administration per eye to lower IOP in patients with OAG and OHT.

Selective laser trabeculoplasty (SLT), a nonpharmacological therapeutic option to lower IOP in patients with OAG or OHT,^2,3,8,9^ can be used as primary therapy in some patients (e.g., those with intolerance or at high risk for nonadherence to IOP-lowering ophthalmic solutions), or in conjunction with topical ocular medications.^2,3,8–10^ However, whether PGA-containing ophthalmic solutions are effective following SLT remains controversial, as some studies have reported less IOP lowering when SLT was used with topical PGAs,^11,12^ whereas others have reported no diminution of IOP-lowering efficacy.^13,14^ Recently published studies have also shown that the effectiveness of SLT is not sustained in many patients,^3,15–23^ but whether those patients can benefit from treatment with the bimatoprost implant (as opposed to topical bimatoprost instillation) has not yet been investigated in a prospective controlled study.

Beagle dogs are commonly used to evaluate the effects of ophthalmic solutions and sustained drug delivery systems,^5, 24–31^ and several other groups have used normotensive dogs to evaluate the IOP-lowering potential of PGAs and other agents.^32–39^ Moreover, the bimatoprost implant was previously shown to effectively lower IOP in a dose-dependent fashion in normotensive beagle dogs.^40^ In the current study, the IOP-lowering effects of SLT were evaluated in normotensive beagle dogs, and the efficacy of the intracameral bimatoprost implant post-SLT was assessed.

## Methods

### Study design

This preclinical study was conducted at Allergan (Irvine, CA), as per the internationally accepted standard of the 3Rs: Reduction, Refinement, and Replacement.

The study adhered to the Association for Research in Vision and Ophthalmology (ARVO) Statement for the Use of Animals in Ophthalmic and Vision Research and was approved by Allergan's Institutional Animal Care and Use Committee before initiation. Veterinary care and oversight were provided throughout the study to ensure appropriate animal care, in compliance with United States Animal Welfare Act regulations in a program accredited by the American Association for Accreditation of Laboratory Animal Care.

### Animals

Eleven normotensive female beagle dogs (Covance Research Products, Denver, PA) that were naive to IOP-lowering treatment, weighed 6–12 kg, and were 7–8 years old were acclimated to weekly handling for 3 months, including 1 month of training with weekly IOP examinations by the same handlers, so that IOP measurements could be performed without topical or general anesthesia during the study period.

Animals were pair-housed in large canine housing units in an environmentally controlled facility providing a daily 12-h light/12-h dark cycle (lights were on from 6 AM to 6 PM). Temperature, humidity, and air flow in the animal rooms were maintained as per facility standard operating procedures and monitored by the Edstrom Watchdog computer system. Animals received 25–40 g of a canine diet (Canine 5006 or 5007, PMI Nutrition, Shoreview, MN) per kg of body weight daily. Water treated by reverse osmosis was provided *ad libitum* through an automatic watering system. Each animal was monitored throughout the study for any signs of pain or distress, and gross ocular examinations were performed daily.

### SLT procedure

One eye per animal was randomly selected as the study eye to receive SLT treatment, whereas the fellow eye served as control (SLT-naive). Animals were anesthetized with a combination of intramuscular ketamine 5 mg/kg (Putney, Inc., Portland, ME) and dexmedetomidine 0.025 mg/kg (Dexdomitor, Putney, Inc.). Following administration to the study eyes of 1−2 drops of topical pilocarpine hydrochloride 4% (Akorn Animal Health, Lake Forest, IL) to constrict the pupil and facilitate visualization of the trabecular meshwork, and 1 drop of proparacaine hydrochloride 0.5% (Akorn Animal Health) to numb the eye before the SLT procedure, a Hwang-Latina 5.0 SLT with flange lens (Ocular Instruments, Bellevue, WA) was used in conjunction with Goniovisc 2.5% (Hub Pharmaceuticals, Rancho Cucamonga, CA) to visualize the iridocorneal angle.

**FIG. 1.**
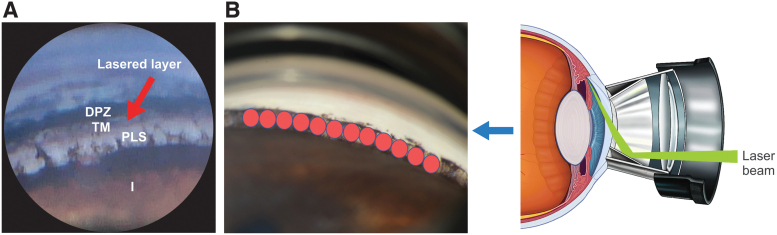
Selective laser trabeculoplasty involved 2 sessions during which the nasal or temporal area (180° each) of the trabecular meshwork **(A)** was treated with adjacent/nonoverlapping laser spots **(B)** to cover the entire 360°. DPZ, deep pigmented zone; I, iris; PLS, pectinate ligament strands; TM, light (bluish) pigmented zone of trabecular meshwork.

The laser beam (Novus Spectra; Lumenis Inc., San Jose, CA) was focused on the trabecular meshwork (Fig. 1), with the energy setting (0.9 mJ) permitting visualization of intermittent small cavitation bubbles. Two SLT treatment sessions (180°/session) were scheduled 2 weeks apart to allow healing and recovery; the nasal or temporal area (180° each) of the trabecular meshwork was treated with adjacent/nonoverlapping laser spots to cover the entire 360° of the meshwork. There was some variation in the total number of laser spots, depending on the size of the eye.

Throughout the procedure, including pre- and post-treatment, animals were monitored by veterinary professionals. Before full recovery and return to housing, the animals were injected with atipamezole 0.25 mg/kg (Antisedan, Orion Corp., Espoo, Finland) to reverse the effects of dexmedetomidine. To alleviate pain and inflammation from day 0 (SLT) to day 3, oral carprofen 4.4 mg/kg (Putney, Inc.) was given once daily and the SLT-treated eye received 1 drop each of ketorolac tromethamine 0.4% (Acular LS, Allergan, an AbbVie company) and prednisolone acetate 1% (Pred Forte, Allergan, an AbbVie company). Topical ophthalmic gatifloxacin 0.5% (Zymaxid, Allergan, an AbbVie company) was applied twice daily from day 0 to day 3 to prevent infection.

### Bimatoprost implant administration

Although the 10-μg dose strength is the approved dose strength in humans, the higher, 15-μg dose strength was used for this study because the anterior chamber volume of the dog is ∼3-fold larger than in humans.^41–43^ A single 15-μg bimatoprost implant was administered intracamerally in both eyes of all animals after a post-SLT follow-up period of 6–10 weeks (intended to allow IOP to return to baseline level).

Animals were first sedated with a combination of intramuscular ketamine 5 mg/kg and dexmedetomidine 0.025 mg/kg, and then prepared for administration of the bimatoprost implant according to standard practice for intracameral injections: after ocular instillation of a broad-spectrum topical antibiotic (gatifloxacin 0.5%) and topical anesthetic (proparacaine hydrochloride 0.5%), the conjunctival surface was irrigated with povidone–iodine 5% ophthalmic solution (Alcon, Fort Worth, TX). This was followed by a saline wash and placement of a lid speculum.

A single-use, prefilled 27-gauge applicator system was used for implant administration. The eye was visualized through an operating microscope (Leica F40, Leica Microsystems, Wetzlar, Germany) and stabilized by grasping the conjunctiva with toothed ophthalmic forceps. The needle entered the anterior chamber through the clear cornea, adjacent the limbus, and advanced 3–4 mm in the anterior chamber, parallel to the iris plane. The implant was then gently deployed by slowly depressing the actuator button (while maintaining the insertion angle unchanged) until an audible click was noted, and microscopic visualization was used to confirm that the implant was properly released. After retraction of the applicator, pressure was applied to the injection site for a few seconds to prevent leakage and allow proper sealing, after which 1 drop of topical gatifloxacin 0.5% was administered.

### Assessments and outcomes

IOP was measured without sedation or topical anesthesia at baseline (≤1 day before the SLT procedure) and weekly post-SLT for 6–10 weeks in all animals, using a TonoVet veterinary rebound tonometer (Icare USA, Raleigh, NC). Following bimatoprost implant administration, IOP was measured on day 3, then weekly for the first 4 weeks, and every 2 weeks up to week 42, based on clinical evidence indicating that the IOP-lowering effect of the bimatoprost implant can last up to a year in some patients.^4,7,44,45^ For each animal, IOP was measured at the same time of day (i.e., 9 AM ±1 h) throughout the study; 3 measurements (each based on 6 consecutive readings automatically obtained and averaged by the tonometer) were taken at each time point, averaged, and used for analysis.

Slit-lamp examination was performed at 2, 6, and 8 weeks postadministration of the bimatoprost implant to assess and grade conjunctival hyperemia (0, none; 1, trace; 2, mild; 3, moderate; 4, severe),^46^ anterior chamber cells (0, <1; 0.5+, 1–5; 1+, 6–15; 2+, 16–25; 3+, 26–50; 4+, >50),^47^ and anterior chamber flare due to inflammation (0, none; 1, faint; 2, moderate with clear iris and lens details; 3, marked with hazy iris and lens details; 4, intense with fibrin or plastic aqueous).^48^ Potential adverse events (AEs) and other examination findings were recorded as well.

The main outcome measures were mean IOP, the mean change in IOP from baseline, and mean percent change in IOP from baseline in SLT-treated and fellow SLT-naive control eyes, both in the 6–10 weeks after the SLT procedure, and up to 42 weeks after bimatoprost implant administration. A *post hoc* analysis of SLT responders was performed, in which animals were categorized as responders if they exhibited peak IOP reductions from baseline ≥3 mmHg or ≥15% lasting more than 1 week (in either eye) after the SLT procedure, or nonresponders if peak IOP reductions from baseline were <3 mmHg or <15%.

### Statistical analyses

Analyses were performed using R statistical analysis software available at http://cran.r-project.org. A linear mixed-effects model was used to evaluate the treatment effect of bimatoprost implant in both SLT-treated and fellow SLT-naive eyes. Multiple comparisons with Tukey's correction were used to evaluate between-treatment differences at each time point. *P* < 0.05 was considered statistically significant.

## Results

### IOP change in SLT-treated eyes

Eleven normotensive beagle dogs, naive to IOP-lowering treatments, received SLT in one eye. The total (standard deviation [SD]) SLT spots on average per treated eye was 217.5 (38.2). At baseline, mean (SD) IOP was 19.3 (1.9) and 19.7 (2.5) mmHg in the eyes to be SLT treated and fellow (untreated control/SLT-naive) eyes, respectively.

SLT produced similar IOP lowering in SLT-treated and fellow SLT-naive eyes. Mean (SD) IOP at peak reduction was 14.2 (3.5) mmHg in SLT-treated eyes, compared with 14.6 (3.0) mmHg in fellow SLT-naive eyes. The corresponding mean (SD) peak IOP reductions were 5.2 (4.5) mmHg and 5.1 (4.4) mmHg, with mean peak percentage IOP reductions of 25.6% and 24.0% in SLT-treated and fellow SLT-naive eyes, respectively.

**FIG. 2.**
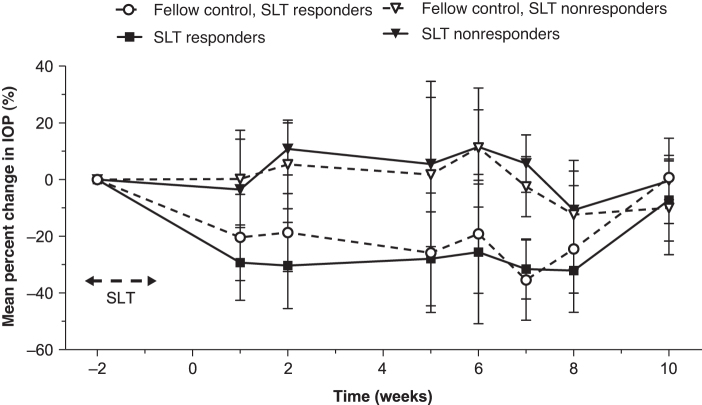
Mean percentage change in IOP from baseline in SLT responder and nonresponder eyes. Data are expressed as mean (SD). IOP, intraocular pressure; SD, standard deviation; SLT, selective laser trabeculoplasty.

Overall, post-SLT IOP had returned to baseline levels between weeks 6 and 10 in 9 of the 11 SLT-treated eyes. The responder analysis demonstrated that 6 (54.5%) animals were SLT responders and 5 (45.5%) were SLT nonresponders. Mean percentage IOP changes from baseline in these subgroups is shown in Fig. 2. IOP returned to baseline between weeks 6 and 10 in 4 of the 6 responders, whereas 2 continued to exhibit IOP reduction ≥3 mmHg up to the time of bimatoprost implant administration.

Among SLT responders, the mean (SD) peak IOP reduction was 7.9 (3.1) mmHg and 7.5 (2.8) mmHg in SLT-treated and fellow SLT-naive eyes, with mean peak percentage IOP reductions of 39.4% and 36.9%, respectively. Among SLT nonresponders, the mean (SD) peak IOP reduction was 1.9 (3.8) mmHg and 2.2 (4.3) mmHg in the SLT-treated and fellow SLT-naive eyes, with mean peak percentage IOP reductions of 9.1% and 8.5%, respectively.

### IOP change in bimatoprost implant-treated eyes

**FIG. 3.**
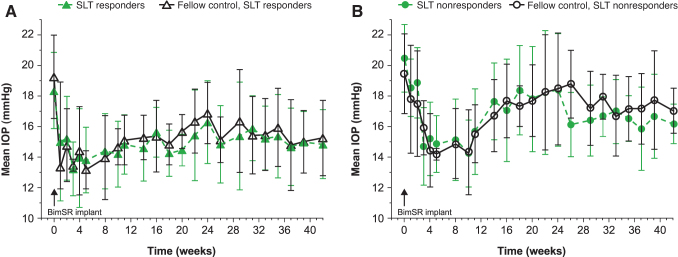
Mean IOP in SLT-treated and fellow SLT-naive eyes of **(A)** SLT responders and **(B)** SLT nonresponders following administration of the bimatoprost implant. Data are expressed as mean (SD). BimSR, bimatoprost sustained-release; IOP, intraocular pressure; SD, standard deviation; SLT, selective laser trabeculoplasty.

The bimatoprost implant had a similar IOP-lowering effect in both SLT-treated and fellow SLT-naive eyes (Fig. 3). The mean (SD) peak IOP reduction following administration of the implant was 6.7 (2.1) mmHg and 7.0 (1.7) mmHg in SLT-treated and fellow SLT-naive eyes, with mean (SD) percentage peak IOP reductions of 34.4% (8.5%) and 35.7% (5.9%), respectively. No statistically significant differences in bimatoprost implant IOP-lowering efficacy were found between SLT-treated and fellow SLT-naive eyes at any time points (*P* > 0.05). In addition, IOP lowering following bimatoprost implant administration was similar whether eyes responded to SLT (*n* = 6) or not (*n* = 5).

Based on mean IOP data, no statistically significant differences in bimatoprost implant IOP-lowering efficacy were observed between SLT responders and SLT nonresponders at any time points (*P* > 0.05) (Fig. 3). Based on mean percent change in IOP data, a statistically significant difference was observed between these groups at 10 weeks (*P* < 0.05), but no other time points. Notably, IOP had not returned to baseline levels at week 42.

### Safety

No SLT-related AEs were reported in any eyes at any time points. Following administration of the bimatoprost implant, all eyes had constricted pupils, which is similar to previous observations in dogs receiving topical PGAs^49^ and is due to F prostaglandin receptors' presence on the iris sphincter muscle. Bimatoprost implant-related AEs included faint anterior chamber flare (1+ in 2 animals) that could have been due to the injection procedure, as well as trace (1 animal) and mild (4 animals) ocular hyperemia (most common ocular AE associated with topical ocular bimatoprost^50^). Both AEs resolved after a 3-day course of topical ketorolac tromethamine 0.5% applied twice daily.

No serious AEs or AEs other than those previously reported^40^ were observed; there was no evidence of photophobia, conjunctival hemorrhage, dry eye, eye irritation, IOP increase, or iritis, for example.

## Discussion

The current prospective study evaluated the IOP-lowering effects of SLT in a canine model, and assessed the efficacy of the intracameral bimatoprost implant post-SLT. Results indicated that SLT can be successfully performed to lower IOP in normotensive beagle dogs. While the effects did not appear to last as long as in human patients with glaucoma,^10,51–53^ some animals did respond better to SLT than others, consistent with clinical observations in humans. In our model, unilateral SLT produced similar mean peak IOP reductions from baseline in SLT-naive and SLT-treated eyes, even when SLT-treated eyes were categorized as SLT responders or SLT nonresponders. Consistent with these findings, some clinical studies of unilateral SLT treatment have also reported IOP lowering in the fellow untreated eye, although to variable extents.^54–58^

Considering that (1) SLT produced similar IOP lowering in treated and untreated eyes, (2) IOP lowering in those eyes was sustained for ≥6 weeks, and (3) no inflammation was observed after the post-SLT 3-day course of anti-inflammatory treatment, it is unlikely that the SLT-induced IOP lowering reported herein was due to inflammation.^59^

Since eyes did not receive concomitant topical IOP-lowering therapy, the possibility of pharmacologic crossover effects between eyes (previously mentioned by McIlraith et al.) does not apply.^57^ Treatment with SLT has, however, been reported to induce the release of vasoactive agents and chemokines [e.g., interleukin (IL)-1α, IL-1β, and tumor necrosis factor-α] that can also act as growth factors in the trabecular meshwork to promote macrophage recruitment, matrix metalloproteinase (MMP)/gelatinase release, and repopulation of the trabecular meshwork with functional cells. Therefore, IOP reduction in the fellow eye could be due to systemic effects of the released vasoactive agents and chemokines, as suggested by Rhodes et al.^56^

Notably, bilateral administration of the intracameral bimatoprost implant effectively lowered IOP in SLT-treated eyes, regardless of response to SLT, as well as fellow SLT-naive eyes.^11,12,60^ By the time the bimatoprost implant was administered bilaterally, the mean IOP of eyes pretreated with SLT had returned to baseline levels and was similar to the mean IOP of fellow SLT-naive eyes in the majority of the animals, which could potentially explain why no difference in efficacy was observed between SLT-treated and fellow SLT-naive eyes following administration of the bimatoprost implant.

Although this study did not address the possibility that administration of the bimatoprost implant in 1 eye might produce IOP lowering in the fellow eye, as appears to be the case for SLT, earlier studies of a single bimatoprost implant in beagle dogs without prior SLT demonstrated no bilateral IOP-lowering effect of the bimatoprost implant with unilateral placement; noticeable differences in IOP lowering were indeed observed between eyes that received the implant (8-, 15-, and 30-μg dose strengths) and untreated fellow eyes until ∼3 months postadministration (Allergan, an AbbVie company; data on file). In addition, pharmacokinetic and pharmacodynamic assessments showed no detectable systemic exposure to bimatoprost or bimatoprost acid at any of the dose strengths evaluated (i.e., 8-, 15-, 30-, and 60-μg).^40^

The current study demonstrated that IOP lowering was sustained for 42 weeks following administration of the bimatoprost implant. In clinical trials, the intracameral bimatoprost lowered IOP well beyond cessation of drug release by the implant.^7^ A proposed mechanism of action for the long-term IOP lowering involves significant upregulation of MMP activity that might produce sustained tissue remodeling in both the trabecular meshwork and uveoscleral pathway (American Glaucoma Society 31st Annual Meeting. 2021). Lee et al.^24^ also suggested another mechanism in which the bimatoprost implant decreases episcleral venous pressure (EVP) in addition to the known PGA-induced increase in both uveoscleral and conventional outflow facilities. The effects of the bimatoprost implant on the trabecular meshwork, uveoscleral pathway, and EVP could explain the long-term effect on IOP.

These experiments in dogs showed that the intracameral bimatoprost implant can lower IOP post-SLT, which provides confidence that a similar effect could occur in humans. Patients with prior SLT treatment were included in 2 phase 3 studies of the bimatoprost implant (ARTEMIS 1, NCT02247804; ARTEMIS 2, NCT02250651), and preliminary data from a retrospective, *post hoc*, pooled analysis of those studies indicated that the effects translated well; the bimatoprost implant lowered IOP in both SLT-naive and SLT-treated human eyes, regardless of response to prior SLT (38th Congress of the European Society of Cataract & Refractive Surgeons, 2020: ESCRS Paper ID FP-453877; American Society of Cataract and Refractive Surgery Symposium on Cataract, IOL, & Refractive Surgery, 2020: ASCRS Paper ID 61926; Saudi Ophthalmology Virtual Symposium, 2021).

Future prospective, clinical studies should also be conducted to confirm the aforementioned preliminary retrospective, clinical findings, as SLT is often used as first-line therapy in patients with glaucoma, and yet medical treatment of IOP is eventually needed in many of those patients.

In the current study, the bimatoprost implant was well tolerated and AEs were consistent with those previously reported in a pharmacokinetic/pharmacodynamic study of the implant in normotensive beagle dogs.^40^ While IOP lowering was sustained for 42 weeks following administration of the bimatoprost implant, cases of trace-to-mild conjunctival hyperemia were resolved after a 3-day treatment, suggesting that conjunctival hyperemia was likely due to application of ophthalmic povidone–iodine during the preparation for implant administration, as previously reported.^4^

A potential limitation of this study was the small sample size and use of a normotensive animal model. However, SLT has been shown to lower IOP in glaucoma patients with normal IOP^61–65^ and, as mentioned above, normotensive dogs have been used by several groups to evaluate the IOP-lowering potential of various pharmacological agents, as well as sustained drug delivery systems. Moreover, some of the animals evaluated herein responded better than others to SLT, as is the case in humans, supporting the model. It is also worth noting that the IOP-lowering effect of SLT in dogs was shorter than that reported in humans (especially newly diagnosed patients without any prior glaucoma therapy).^51,52^ However, the majority of patients evaluated in clinical practice will have used glaucoma eyedrops before SLT treatment, and the duration of SLT IOP-lowering effects can vary considerably in human patients.

## Conclusions

This was the first study to prospectively evaluate the efficacy of a PGA-containing, sustained-release implant following SLT treatment. Results from the canine model suggest that intracameral administration of the bimatoprost implant can effectively lower IOP in eyes previously treated with SLT, regardless of response to SLT.
